# Awake craniotomy for drug-resistant non-oncological epilepsy: a systematic review of feasibility, reported safety, and seizure outcomes

**DOI:** 10.1186/s12893-026-04115-4

**Published:** 2026-08-10

**Authors:** Mohammad Mofatteh, Mohammad Sadegh Mashayekhi, Sho Giersztein, Arad Iranmehr, Maysam Alimohamadi, Davide Giampiccolo, Hugues Duffau, Aaron A. Cohen-Gadol

**Affiliations:** 1 School of Medicine, Dentistry and Biomedical Sciences, Queen’s University Belfast, Belfast, UK; 2 Department of Neurosurgery, Neuro International Collaboration (NIC), London, UK; 3 Faculty of Medicine, Division of Neurosurgery, University of Ottawa, Ottawa, ON Canada; 4 Department of Neurosurgery, Neuro International Collaboration (NIC), Vancouver, Ottawa, ON Canada; 5 Faculty of Medicine and Health Sciences, University of East Anglia, Norwich, UK; 6 Department of Neurosurgery, Sina Hospital, Tehran University of Medical Sciences, Tehran, Iran; 7 National Hospital for Neurology and Neurosurgery, Queen Square, London, UK; 8 Department of Neurosurgery, Gui de Chauliac Hospital, Montpellier University Medical Center, Montpellier, France; 9 ATLAS Institute of Brain and Spine and Neurosurgical Atlas, Los Angeles, CA USA

**Keywords:** Awake craniotomy, Awake brain surgery, Brain mapping, Epilepsy, Seizure

## Abstract

**Introduction:**

Complete resection of the epileptogenic zone offers the best chance of seizure control in patients with drug-resistant epilepsy. However, surgical management remains challenging when the epileptogenic zone is located within or adjacent to eloquent brain regions. Awake craniotomy (AC), combined with intraoperative functional mapping, has the potential to maximize resection while preserving neurological function. Despite its increasing use, evidence regarding its safety and efficacy in epilepsy surgery remains limited. This study aimed to systematically evaluate the feasibility, reported safety, and reported seizure outcomes of awake craniotomy in patients undergoing surgery for drug-resistant non-oncological epilepsy.

**Methods:**

A systematic literature search was conducted in PubMed, Scopus, and Web of Science databases in accordance with PRISMA guidelines.

**Results:**

Seven retrospective studies comprising 231 patients from six countries were included; 120 patients underwent AC. Four studies (57.1%) included mixed adult and pediatric populations and three (42.9%) included adults only; however, none provided age-stratified seizure or complication outcomes, precluding separate pediatric analysis. Reported seizure duration ranged from 2 to 39 years. The frontal lobe was the most frequently involved region (*n* = 52; 43.3%), followed by the temporal lobe (*n* = 27; 22.5%), and cortical dysplasia was the most common underlying pathology (*n* = 30; 25.0%). Language and sensorimotor functions were the most commonly mapped eloquent areas, each reported in five studies (71.4%). Seizure outcomes were predominantly assessed using the Engel classification. Complete seizure freedom (Engel class I) was reported in 69 of 120 AC patients (57.5%); a random-effects single-arm synthesis estimated an Engel I proportion of 59.3% (95% CI: 46.3–71.2%; I2 = 31.9%). Comparisons with surgery under general anesthesia (GA) were available in only two non-randomized studies and should be interpreted cautiously. No study reported conversion from AC to GA. Postoperative neurological deficits were largely transient, with a low reported incidence of permanent morbidity. Owing to substantial differences in patient selection, surgical indications, and study design, these findings should be considered exploratory and should not be interpreted as demonstrating comparable seizure outcomes or treatment effectiveness.

**Conclusions:**

In carefully selected patients with drug-resistant non-tumor epilepsy involving or adjacent to eloquent cortex, AC with intraoperative functional mapping appears feasible and may help preserve neurological function while enabling tailored resection. The current evidence base remains limited by retrospective designs, heterogeneous populations and techniques, inconsistent outcome reporting, and sparse comparative data; therefore, efficacy and equivalence with surgery under general anesthesia should not be inferred.

**Supplementary Information:**

The online version contains supplementary material available at https://doi.org/10.1186/s12893-026-04115-4.

## Introduction

Since the introduction of localizing epileptic focus concept by Sir Victor Horsley in 1886 and its expansion by the pioneering work of Wilder Penfield and others more than 80 years ago, neurosurgical indications of awake craniotomy (AC) have been broadened beyond epilepsy to include primary tumors, metastases, and arteriovenous malformations [1–12]. A further combination of AC with direct cortical and subcortical stimulation has been used to map and monitor neurological functions, which resulted in the expansion of our understanding of brain function [13].

As a well-established, evidence-based treatment, surgery for epilepsy aims to maximize seizure control while minimizing neurological complications by identifying and resecting epileptogenic zones [14–21]. However, neurosurgical management of medically refractory seizures is challenging when the epileptogenic zone is located within or near the eloquent areas, as a balance should be achieved regarding the resection of the lesion while minimizing potential postoperative deficits by preserving eloquent areas [22–24]. AC, where the patient is awake for the entire or part of the operation, enables intraoperative monitoring of eloquent areas to preserve their neurological function [25–31].

While other studies have shown that AC for tumors can result in overall favorable outcomes, with increased survival, lower neurological deficits, faster recovery, shorter hospitalization, and better psychological sequelae, even in resource-limited settings [32–38] differences in subcortical organizations, neuroplasticity, functional reorganization, and anatomical changes can render non-tumor epilepsy surgery a different field [39–42]. Therefore, the usage of AC for non-tumor epilepsy remains understudied.

In this study, we aimed to better understand the feasibility, reported safety, and seizure outcomes of awake craniotomy combined with intraoperative functional mapping in patients undergoing epilepsy surgery for non-oncological epileptogenic lesions. Throughout this review, non-tumor epilepsy refers to epilepsy managed primarily for seizure control rather than oncological tumor resection. Developmental epileptogenic lesions, including focal cortical dysplasia, malformations of cortical development, and dysembryoplastic neuroepithelial tumors (DNETs), were included when surgery was undertaken primarily for treatment of drug-resistant epilepsy rather than tumor management.

## Methods

### Inclusion and exclusion criteria

We used a search strategy that was described previously [34–38]. Articles were included in our systematic review if they met the following criteria: (1) original research article, (2) English language only, (3) involved human subjects only, (4) AC for the treatment of epilepsy as the main indication, (5) sufficient data could be extracted from an article on publicly available databases. Our exclusion criteria were defined as: (1) studies which had a mixture of epileptic and non-epileptic patients, (2) epilepsy was secondary to other pathologies, such as epileptogenic glioma, (3) AC and GA techniques were used without the possibility of individual patient data differentiation, (4) sufficient data could not be extracted from the article, (5) case reports or case series including fewer than 4 patients (small series of 4 to 5 patients were retained given the rarity of the topic but interpreted cautiously), (6) reviews, book chapters, and abstracts. Studies were excluded when awake craniotomy was performed primarily for resection of primary or metastatic intracranial neoplasms (e.g., diffuse gliomas or brain metastases), even if seizures were present.

### Data source and search strategy

This systematic review was conducted following the Preferred Reporting Items for Systematic Reviews and Meta-Analysis (PRISMA) guidelines [43] to identify published literature on AC for the treatment of epilepsy. This review was not prospectively registered (e.g., PROSPERO), which represents a methodological limitation. Nevertheless, eligibility criteria, search strategy, study selection, data extraction, and quality assessment were predefined before study screening commenced, and the review was conducted in accordance with PRISMA recommendations. PubMed, Scopus, and Web of Science were searched from database inception to 22 July 2024 using a deliberately sensitive search strategy centered on awake craniotomy terminology. Because awake craniotomy for epilepsy represents an uncommon indication and relevant studies are frequently indexed according to surgical technique rather than underlying disease, broad awake surgery terms were selected to maximize retrieval. During preliminary scoping searches, incorporation of epilepsy-specific keywords substantially reduced identification of eligible publications. Therefore, potentially relevant epilepsy studies were identified during title/abstract screening and confirmed by full-text review using predefined eligibility criteria. The following Boolean terms were used for the search: (“awake craniotomy” OR “awake brain surgery” OR “awake neurosurgery” OR “awake brain mapping” OR “awake tumor resection” OR “craniotomy while awake”). Supplementary Table 1 demonstrates details of search terms for each database.

### Article selection process

Following removal of duplicates, two reviewers independently screened titles and abstracts. Studies describing awake craniotomy performed primarily for oncological indications, vascular lesions, or other non-epilepsy conditions were excluded at this stage. Full-text review was subsequently undertaken to confirm that the primary indication for surgery was medically refractory epilepsy. Studies in which epilepsy occurred secondary to diffuse glioma, metastatic disease, or mixed oncological cohorts without extractable epilepsy-specific data were excluded.

### Data extraction

At least two authors independently extracted data from each selected paper in the final review. The following data of interest were extracted from the final articles: first author, publication year, title, journal name, the country where the study was conducted, study objectives, inclusion and exclusion criteria for AC, study period, study design, single/multi-center, condition treated, anesthesia protocol, primary sedation and analgesia, follow-up period, total patient number, whether AC and GA were compared directly in the article, patient population (adult/pediatric), patients’ mean/median age, sex, seizure outcome assessment tool, complete seizure resolution, hospitalization length, intraoperative complications, seizure duration, epilepsy hemisphere, epilepsy lobe, eloquent area lesion and mapping, AC extent of resection, conversion to GA, pre-operative neurological symptoms, post-operative complications and neurological deficits, and the main outcome reported. The Microsoft Excel Spreadsheet was used to manage independently extracted data (version 2016; Microsoft, Redmond, WA, USA).

### Data synthesis and risk-of-bias assessment

Given the small number of included studies and the clinical heterogeneity of pathologies, age groups, mapping techniques, and reporting methods, the primary synthesis was descriptive. To address the main quantitative outcome, a single-arm random-effects meta-analysis of Engel class I seizure freedom after AC was performed using a logit transformation with continuity correction for studies with 100% event rates. Between-study heterogeneity was summarized using I2. Comparative AC-versus-GA findings were interpreted narratively because only two non-randomized studies included GA comparators and selection bias could not be excluded. Outcomes for intraoperative seizures, postoperative neurological deficits, quality of life, and postoperative psychological or social adaptation were summarized descriptively because reporting was inconsistent across studies. Risk of bias was assessed using a modified Newcastle-Ottawa Scale [44] adapted for retrospective surgical cohorts and case series, with emphasis on selection, comparability, outcome ascertainment, follow-up adequacy, and reporting completeness.

## Results

### Study selection

**Fig. 1:**
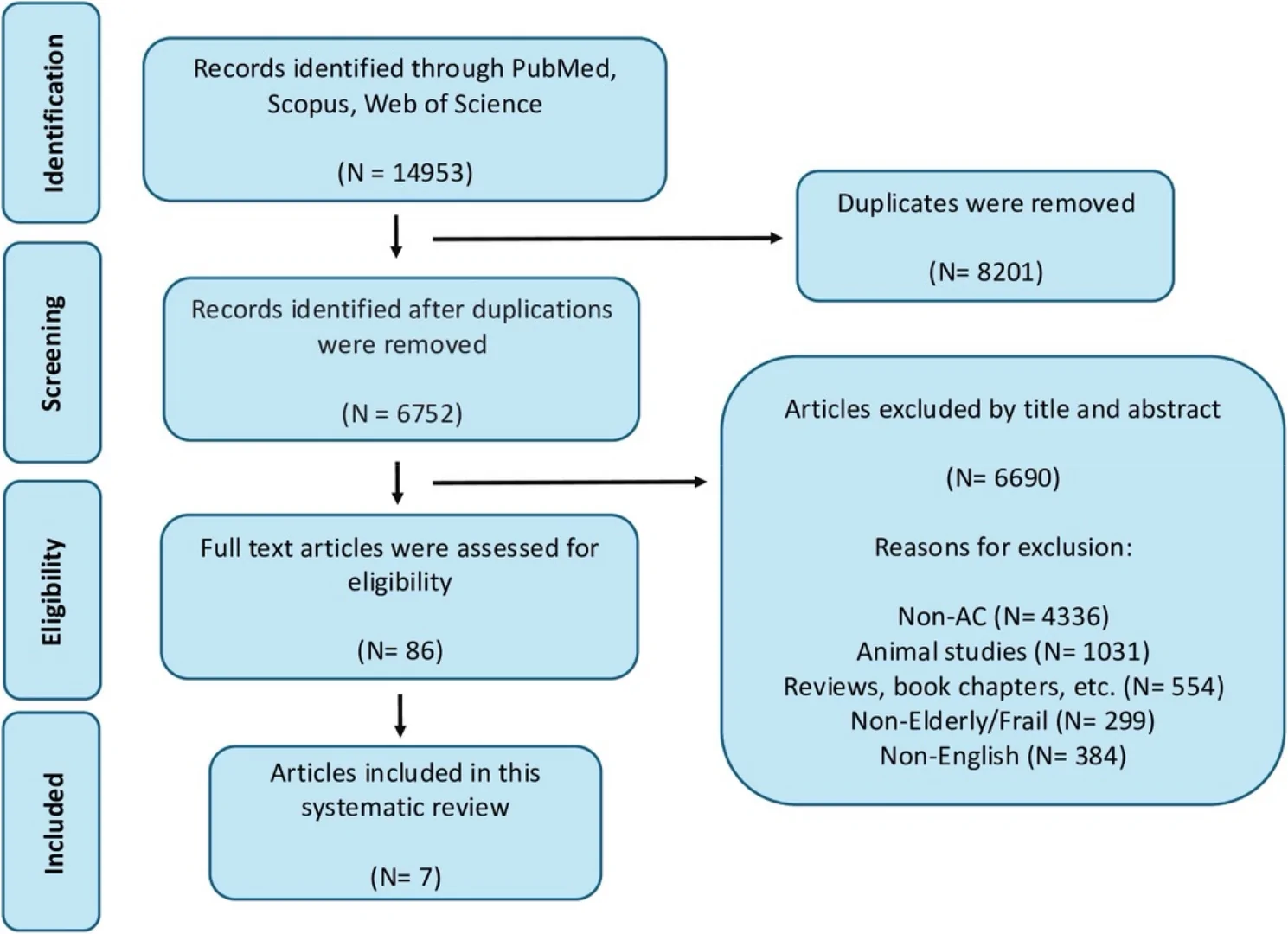
Preferred reporting items for systematic reviews and meta-analyses (PRISMA) flowchart demonstrates the current study’s identification, screening, eligibility, and inclusion process. AC, awake craniotomy

According to the PRISMA flow diagram, a total of 14,953 records were initially identified across PubMed, Scopus, and Web of Science. After removal of duplicate records (*n* = 8,201), 6,752 articles remained for title and abstract screening. Of these, 6,690 articles were excluded based on the predefined inclusion and exclusion criteria. The primary reasons for exclusion were studies not related to AC (*n* = 4,336), animal studies (*n* = 1,031), review articles and other non-original publications (*n* = 554), studies not focusing on the target population (*n* = 299), and non-English publications (*n* = 384). For studies meeting multiple exclusion criteria, only one reason was recorded. A total of 86 full-text articles were assessed for eligibility, of which 79 were excluded following full-text review. Ultimately, 7 studies met the inclusion criteria and were included in the final systematic review (Fig. 1).

A summary of the included studies is presented in Table 1. Of the seven articles included [45–51], two (28.6%) were from Sweden [48, 50], and one (14.3%) each from the USA [45], South Korea [46], France [47], Bulgaria [49], and Japan [51].

**Table 1:** A summary of studies included

Study		Journal	Country	Objective	Inclusion and Exclusion Criteria for AC
Cohen-Gadol et al., 2003 [45]	Nonlesional central lobule seizures: use of awake cortical mapping and subdural grid monitoring for resection of seizure focus	Journal of Neurosurgery	USA	To describe an alternative surgical approach for treating medically refractory non-lesional perirolandic epilepsy.	NS
Kim et al., 2011 [46]	Resection frequency map after awake resective surgery for non-lesional neocortical epilepsy involving eloquent areas	Acta Neurochirurgica	South Korea	To assess the efficacy and risk of AC for non-lesional epilepsy involving the eloquent areas or their adjacent areas and to generate a resection frequency map.	Inclusion: NS. Exclusion: patients without preoperative and postoperative MR data with a T1-weighted three-dimensional spoiled gradient
Korkar et al., 2020 [47]	Awake craniotomy for epilepsy surgery on eloquent speech areas: A single-center experience	Epileptic Disorders	France	To report the efficacy of AC with cortical language cortex mapping to resect non-glioma epileptic zones with regards to seizure control and functional outcome.	Inclusion: the epileptogenic zone close to eloquent speech areas. Exclusion: children less than 14 years of age, patients with morbid obesity, severe dysphasia, psychiatric history, emotional instability or severe anxiety disorder, impaired cognition or intellectual disability.
Vigren et al., 2020 [48]	Experiences of awake surgery in non-tumor epilepsy in eloquent localisations	Clinical Neurology and Neurosurgery	Sweden	To demonstrate the safety and efficiency of AC in patients typically excluded from epilepsy surgery.	NS
Minkin et al., 2021 [49]	Awake Epilepsy Surgery in Patients with Focal Cortical Dysplasia	World Neurosurgery	Bulgaria	To evaluate the benefits of AC in drug-resistant epilepsy patients caused by focal cortical dysplasia in eloquent areas.	Inclusion: consecutive patients with drug-resistant seizures (continuous seizures despite two antiepileptic drugs for one year) involved eloquent cortex during the study period.
Vigren et al., 2023 [50]	Awake craniotomy in epilepsy surgery includes previously inoperable patients with preserved efficiency and safety	International Journal of Neuroscience	Sweden	To investigate the safety and efficacy of AC in epilepsy surgery and focus on the possibility of widening surgical indications with AC.	Inclusion: patients with the presence of a putative epileptogenic zone adjacent to an eloquent area, fluent Swedish speaker and a normal preoperative anesthesiological assessment. Exclusion: patients with isolated mesial sclerosis as an epileptogenic zone, other conditions, such as pulmonary or heart failure, psychiatric disorders and severe obesity.
Uda et al., 2024 [51]	Awake Craniotomy in Epilepsy Surgery: A Case Series and Proposal for Three Different Scenarios	Brain Sciences	Japan	To report a case series of patients undergoing AC in epilepsy surgery and propose three clinical scenarios defining its indications and roles.	Inclusion: patients undergoing AC for epilepsy surgery between 2014–2023; epilepsy surgery candidates (typically drug-resistant epilepsy after ≥ 2 antiseizure medications). Exclusion: NS

*AC* awake craniotomy, *NS* not specified

### Study characteristics

An overview of patient demographics is presented in Table 2. A total of 231 patients were included across seven studies. Four studies included a mixture of adult and pediatric populations [45–47, 49], whereas three studies focused exclusively on adults [48, 50, 51]. Two retrospective studies reported seizure outcomes following both awake craniotomy and surgery under general anesthesia [49, 50]: across all included studies, 120 patients (51.9%) underwent AC. However, differences in study design, patient selection, underlying pathology, and indications for awake surgery preclude meaningful comparative interpretation.

**Table 2:** An overview of prevalence and demographics of patients from included studies

Study				Number of patients		Mean age (years) ± SD (range) at the surgery		Sex AC		Sex GA	
	Total patient number	Adults (*≥*18 years) or pediatric (* <*18 years)	AC and GA comparison	AC (*n*, %)	GA (*n*, %)	AC	GA	Males (*n*, %)	Females (*n*, %)	Males (*n*, %)	Females (*n*, %)
Cohen-Gadol et al., 2003 [45]	5	Adults and pediatric	No	5 (100%)	0 (0.0%)	28.6 (NS, 16–56)	NA	4 (80.0%)	1 (20.0%)	NA	NA
Kim et al., 2011 [46]	55	Adults and pediatric	No	55 (100%)	0 (0.0%)	26 (NS, 15–51)	NA	31 (56.4%)	24 (43.6%)	NA	NA
Korkar et al., 2020 [47]	17	Adult and pediatric	No	17 (100%)	0 (0.0%)	26.4 (12, 16–40)	NA	10 (58.8%)	7 (41.2%)	NA	NA
Vigren et al., 2020 [48]	4	Adults	No	4 (100%)	0 (0.0%)	32.8 (5.31, 25–37)	NA	0 (0.0%)	4 (100.0%)	NA	NA
Minkin et al., 2021 [49]	95	Adults and pediatric	Yes	14 (14.7%)	81 (85.3%)	27.9 (NS, 14–43)	NS	8 (57.1%)	6 (42.9%)	NS	NS
Vigren et al., 2023 [50]	45	Adults	Yes	15 (33.3%)	30 (66.7%)	39 (NS, NS)	33 (NS, NS)	6 (40.0%)	9 (60.0%)	13 (43.3%)	17 (56.7%)
Uda et al., 2024 [51]	10	Adults and pediatric	No	10 (100%)	0 (0.0%)	26.2 (SD not specified, 14–41)	NA	5 (50.0%)	5 (50.0%)	NA	NA

*NA* not applicable, *NS* not specified, *SD* standard deviation

Among AC patients, 64 (53.3%) were male and 56 (46.7%) were female. The youngest mean age of AC patients was 26 years [46], while the oldest mean age was 39 years [50]. The youngest individual patient reported was 14 years [49, 51], and the oldest was 56 years [45].

Study characteristics are summarized in Table 3. All included studies used a retrospective design. The majority were conducted at a single center [45–49, 51], with one study adopting a multicenter approach [51]. Study periods varied considerably across the included articles, reflecting differences in institutional experience and case volume. AC protocols were heterogeneous; while some studies explicitly used an asleep-awake-asleep technique [49, 50], others did not specify a standardized protocol [45–48, 51]. Similarly, reporting of anesthetic strategies, perioperative antiepileptic drug use, stimulation parameters, and operative duration was inconsistent, with only a subset of studies providing detailed information on sedation regimens and surgical times.

**Table 3:** A Summary of studies characteristics

Study	Study period	Study design	Single/ multi-center	AC protocol	Primary sedation and analgesia	Mean AC operation time ± SD (range)	Follow-up (Mean years, range)
Cohen-Gadol et al., 2003 [45]	1996–2000	Retrospective	Single-center	NS	NS	NS	3.5 years (2.5-5 years)
Kim et al., 2011 [46]	1994–2007	Retrospective	Single-center	NS	Bupivacaine, epinephrine propofol	300 min (155–450 min)	6 years (6-136 months)
Korkar et al., 2020 [47]	2010–2019	Retrospective	Single-center	NS	Levobupivacaine, dexmedetomidine or propofol, remifentanil, hypnosis	NS (mean duration of the awake phase: 75 min, range 50–110 min)	5.7 years (NS)
Vigren et al., 2020 [48]	NS	Retrospective	Single-center	NS	NS	NS	NS (1–3 years)
Minkin et al., 2021 [49]	2009–2018	Retrospective	Single-center	Asleep-awake-asleep	Propofol and remifentanil	NS	6 years (2–10 years)
Vigren et al., 2023 [50]	2016–2020	Retrospective	Single-center	Asleep-awake-asleep	NS	NS	2 years (NS)
Uda et al., 2024 [51]	2014–2023	Retrospective case series	Multi-center	NS	NS	NS	NS

*NS* not specified

Four studies (57.1%) included both adult and pediatric patients [45–47, 49]; however, none provided sufficiently granular age-stratified seizure or complication outcomes, and therefore pediatric and adult outcomes could not be analyzed separately. Follow-up duration also varied between studies, and was not consistently reported, limiting direct comparison of long-term outcomes across cohorts.

Lesion and seizure characteristics are summarized in Table 4. Cortical dysplasia was the most frequently reported pathology, accounting for 30 patients (approximately 25–27%) undergoing AC across the included studies. Among studies that reported seizure duration [45–47], the longest mean duration was 20.2 years [45]. The longest individual seizure duration reported was 39 years [45], whereas the shortest was 2 years [46, 47].

**Table 4:** A summary of seizure characteristics

Study	Epilepsy type (*n*, %)	Seizure duration (Mean, range)	Lesion hemisphere		Lesion location				
			Left (*n*, %)	Right (*n*, %)	Frontal (*n*, %)	Temporal (*n*, %)	Parietal (*n*, %)	Insular (*n*, %)	Other (*n*, %)
Cohen-Gadol et al., 2003 [45]	Non-lesional simple partial seizure (5, 100%)	20.2 years (8–39)	1 (20.0%)	4 (80.0%)	NS	NS	NS	NS	NS
Kim et al., 2011 [46]	Simple partial seizures (4, 7.3%), CPS (7, 12.7%), CPS & secondary generalization (28, 50.9%), generalized tonic-clonic seizures (16, 29.1%)	13 years (2–3)	30 (54.5%)	25 (45.5%)	27 (49.1%)	8 (14.5%)	10 (18.2%)	0 (0.0%)	10 (18.2%)
Korkar et al., 2020 [47]	FCD (4, 23.5%), DNET (2, 11.8%), non-lesional epilepsy (3, 17.6%), tuberous sclerosis (1, 5.9%), hypoxic-ischemic insult (1, 5.9%), other cortical development malformations, e.g., cortical thickening, dysplasia, etc. (6, 35.3%)	15.7 years (2–37)	17 (100%)	0 (0.0%)	7 (41.2%)	5 (29.4%)	0 (0.0%)	5 (29.4%)	0 (0.0%)
Vigren et al., 2020 [48]	Sturge-Weber malformation (1, 25.0%), cortical dysplasia (1, 25.0%), schizencephaly (1, 25.0%), MRI-negative epilepsy (1, 25.0%)	NS	2 (50.0%)	2 (50.0%)	1 (25.0%)	2 (50.0%)	0 (0.0%)	0 (0.0%)	Occipital (1, 25.0%)
Minkin et al., 2021 [49]	FCD (14, 100%)	NS	NS	NS	9 (64.3%)	3 (21.4%)	0 (0.0%)	0 (0.0%)	Fronto-temporo-insular (1, 7.1%), temporo-parieto-occipital (1, 7.1%)
Vigren et al., 2023 [50]	Cortical dysplasia (6, 40.0%), gliosis (5, 33.3%), hemangioma (3, 20.0%), other (1, 6.7%)	NS	9 (60.0%)	6 (40.0%)	2 (13.3%)	7 (46.7%)	3 (20.0%)	0 (0.0%)	Occipital (3, 20.0%)
Uda et al., 2024 [51]	FAS ± FBTCS: 4 (40%) FIAS ± FBTCS: 6 (60%)	NS	7 (70.0%)	3 (30.0%)	6 (60.0%)	2 (20.0%)	0 (0.0%)	1 (10.0%)	Fronto-parietal: 1 (10.0%)

*CPS* complex partial seizures, *DNET* dysembryoplastic neuroepithelial tumors, *FAS* focal awareness seziure, *FBTCS* focal to bilateral tonic clonic seizures, *FCD* focal cortical dysplasia, *FIAS* focal impaired awareness seizures, *NS* not specified*1 patient (5.9%) did not undergo resection

Although the review focused on non-oncological epilepsy surgery, the included studies comprised heterogeneous structural etiologies. Most patients had developmental epileptogenic lesions, including focal cortical dysplasia and malformations of cortical development. One study also included a small number of patients with dysembryoplastic neuroepithelial tumors, in whom surgery was undertaken primarily for seizure control rather than tumor progression [47]. No included study evaluated awake craniotomy performed primarily for diffuse glioma or other oncological indications.

With regard to lesion location, the frontal lobe was the most commonly affected region, reported in 52 patients (43–45%), followed by the temporal lobe in 27 patients (23%). Less frequently involved regions included the parietal and insular lobes. Data from the more recent study by Uda et al. [51] further support this distribution, with a predominance of frontal lobe involvement (60%) and a higher proportion of left hemispheric lesions (70%). However, not all studies reported lesion location or resection extent in detail, limiting direct quantitative comparisons across cohorts.

Operative details are summarized in Table 5. Language mapping [46–50] and sensorimotor mapping [45, 46, 48–51] were the most frequently assessed eloquent functions, each reported in five studies (71.4%). Visual mapping was less commonly performed, being reported in two studies (28.6%) [48, 50]. Direct electrical stimulation was used across all studies to guide intraoperative functional mapping, although the extent and complexity of mapping varied between cohorts.

**Table 5:** A summary of operation details

Study	Eloquent area mapping	Seizure outcome tool	Seizure-free outcome (*n*, %)	Intraoperative complications	Conversion to GA (*n*, %)	Hospitalization length	
						AC	GA
Cohen-Gadol et al., 2003 [45]	Sensorimotor	Engel classification	4 (80.0%)	None	0 (0.0%)	NS	NA
Kim et al., 2011 [46]	Language, Sensorimotor	Engel classification	27 (49.1%)	seizures, loss of cooperativeness, irrecoverable functional deficit	0 (0.0%)	7 days mean (3–24 days)	NA
Korkar et al., 2020 [47]	Language	Engel classification	11 (64.7%)	Expressive dysphasia and distal motor weakness (2, 12.5%)	0 (0.0%)	NS	NA
Vigren et al., 2020 [48]	Language, motor, vision	Engel classification	4 (100%)	NS	0 (0.0%)	NS	NA
Minkin et al., 2021 [49]	Language, motor, somatosensory	Engel classification	10 (71.4%)	Seizure (4, 28.6%)	0 (0.0%)	NS	NS
Vigren et al., 2023 [50]	Language, motor, vision	Engel classification	10 (66.7%)	NS	0 (0.0%)	NS	NS
Uda et al., 2024 [51]	Motor and language mapping (positive/negative cortical and subcortical stimulation)	Engel classification	3/10 (30%) Engel class IA	None significant; minor postoperative deficits (1 sensory deficit, 1 cognitive decline)	0 (0.0%)	NS	NS

*AC* awake craniotomy, *GA* general anesthesia, *NA* not applicable, *NS* not specified

Seizure outcomes were most commonly evaluated using the Engel classification, which was used in the majority of studies [45–51]. Across the included cohorts, complete seizure freedom (Engel class I) was reported in 69 of 120 AC patients (57.5%). In the single-arm random-effects synthesis, the pooled Engel I proportion was 59.3% (95% CI: 46.3–71.2%; I2 = 31.9%), indicating moderate uncertainty and modest between-study heterogeneity. Because only two non-randomized studies included an internal GA comparator, comparisons between AC and GA were not pooled and should be interpreted as hypothesis-generating rather than confirmatory. None of the included studies reported conversion from AC to general anesthesia, supporting procedural feasibility in carefully selected patients.

Technical mapping characteristics and stimulation-related issues are summarized in Table 6. Considerable heterogeneity was observed in mapping techniques and reporting practices. Most studies used awake cortical or functional mapping using direct electrical stimulation (7/7; 100%), although the extent of technical detail varied substantially. Only two studies (28.6%) clearly described an asleep-awake-asleep protocol [49, 50]. Stimulation parameters were not specified in any study (0/7; 0%), and preventive antiepileptic drug strategies were clearly reported in none (0/7; 0%).

**Table 6:** Technical mapping characteristics and stimulation-related issues

Study	Mapping approach / ECoG	Mapped functions	Stimulation-related or intraoperative seizure issues	Key reporting limitation
Cohen-Gadol et al., 2003 [45]	Awake cortical mapping with subdural grid monitoring	Sensorimotor	No intraoperative complications reported	Stimulation parameters and AED prophylaxis not specified
Kim et al., 2011 [46]	Awake resective surgery with intraoperative mapping	Language and sensorimotor	Seizures, loss of cooperativeness, and irrecoverable functional deficit reported, but frequency not fully extractable	Incomplete quantification of stimulation-related complications
Korkar et al., 2020 [47]	Direct cortical mapping during AC	Language	Expressive dysphasia and distal motor weakness reported intraoperatively/postoperatively	AED prophylaxis and stimulation parameters not consistently extractable
Vigren et al., 2020 [48]	Awake mapping; details varied by case	Language, motor, vision	Not specified	Small case series with limited technical reporting
Minkin et al., 2021 [49]	Asleep-awake-asleep AC with direct stimulation	Language, motor, somatosensory	Intraoperative seizure in 4/14 AC patients (28.6%)	Preventive AED strategy not clearly reported in extractable data
Vigren et al., 2023 [50]	Asleep-awake-asleep AC with functional mapping	Language, motor, vision	Not specified	Limited detail on stimulation parameters

*AC* awake craniotomy, *AED* antiepileptic drug, *EcoG* electrocorticography

Intraoperative seizure reporting was heterogeneous. Minkin et al. [49] provided the only fully quantifiable report, documenting intraoperative seizures in 4 of 14 AC patients (28.6%; 95% CI: 8.4–58.1%). Kim et al. [46] listed seizures among complications but did not report a clear denominator. Cohen-Gadol et al. [45] reported no intraoperative complications in their five-patient series. The remaining four studies either did not specify or did not provide extractable data on stimulation-related seizure events. Because of this inconsistency, a pooled intraoperative seizure rate could not be calculated.

Operative outcomes are summarized in Table 7. Across the included studies, postoperative neurological deficits were predominantly transient, with most resolving during follow-up. A small number of permanent deficits were reported, including mild dysarthria (*n* = 1, 0.4%), coordination disturbance of the hand (*n* = 1, 0.4%), and permanent motor dysphasia (*n* = 1, 0.4%). In addition, minor postoperative deficits such as sensory disturbance and cognitive decline were reported in a small proportion of patients in more recent cohorts. Overall, the incidence of permanent neurological morbidity remained low across studies.

**Table 7:** Major operative outcomes reported

Study	Postoperative neurological deficits (*n*, %)	Main outcome
Cohen-Gadol et al., 2003 [45]	Transient hemiparesis (3, 60.0%), transient cortical sensory loss and apraxia (4, 80.0%).	AC for limited resection of the sensorimotor cortex can be performed as an alternative approach to multiple subpial transections in patients with medically refractory perirolandic epilepsy.
Kim et al., 2011 [46]	Permanent mild dysarthria (1, 1.9%), transient weakness of upper extremities (1, 1.9%), transient paraesthesia of hand (1, 1.9%), permanent coordination disturbance of hand (1, 1.9%), transient weakness of lower extremities (1, 1.9%), transient motor dysphasia (3, 5.4%), transient sensory dysphasia (1, 1.9%), permanent motor dysphasia (1, 1.9%)	AC combined with intraoperative brain mapping is a safe and effective treatment option for non-lesional epilepsy involving eloquent areas. Establishing a resection frequency map can provide an objective assessment of neurological risk.
Korkar et al., 2020 [47]	Transient expressive dysphasia (1, 5.9%), transient uncomfortable reading (1. 5.9%), transient expressive dysphasia + distal motor weakness (1, 5.9%), transient right facio-brachial mild weakness (1, 5.9%).	AC can be safely used to help patients, typically candidates for palliative measures, with intractable epilepsy near or in the eloquent language cortex to achieve seizure-free status.
Vigren et al., 2020 [48]	Visual impairment (inferior left quadrant; 1, 25.0%), memory disturbance (2, 50.0%), focal aware seizures (1, 25.0%), anomia (1, 25.0%)	AC combined with intraoperative mapping expands the indications for epilepsy surgery.
Minkin et al., 2021 [49]	None	The rate of complete seizure control in the AC group was comparable to the seizure outcome in the GA group. AC results in a good rate of postoperative seizure control in FCD patients involving the eloquent area in the absence of permanent postoperative neurologic deficit.
Vigren et al., 2023 [50]	Dysphasia (1, 6.7%)	AC for epilepsy surgery is a safe and feasible approach in patients otherwise regarded as inoperable with epileptogenic regions in proximity of eloquent brain structures, resulting in a similar level of seizure control compared to conventional asleep surgery.
Uda et al., 2024 [51]	Mild deficits in 2/10 (20%): Left hand numbness (1, 10%), Mild cognitive decline (1, 10%)	AC is a safe and adaptable technique in epilepsy surgery, enabling individualized surgical strategies across different clinical scenarios with acceptable functional outcomes.

*AC* awake craniotomy, *FCD* focal cortical dysplasia, *GA* general anesthesia, *NS* not specified

Despite variations in study design, patient populations, and surgical techniques, all included studies consistently concluded that AC is a safe and feasible approach for the management of epilepsy in eloquent brain regions. Furthermore, several studies emphasized that awake functional mapping enables maximal resection while preserving neurological function, thereby expanding surgical eligibility to patients who may otherwise be considered inoperable.

Risk of bias was assessed using a modified Newcastle-Ottawa Scale (NOS) for non-randomized studies, with additional attention to reporting completeness for small retrospective case series (Table 8). This approach was chosen because the evidence base consisted predominantly of retrospective surgical cohorts and case series rather than randomized or prospective comparative studies. Overall, the included studies demonstrated a moderate to high risk of bias, primarily due to retrospective design, small sample sizes, heterogeneous eligibility criteria, incomplete reporting of stimulation parameters and perioperative antiepileptic strategies, inconsistent follow-up, and lack of controlled comparisons.

**Table 8:** Risk of bias assessment of included studies

Study	Selection Bias	Comparability	Outcome Assessment	Follow-up Adequacy	Reporting Bias	Overall Risk of Bias
Cohen-Gadol et al., 2003 [45]	High	High	Moderate	Moderate	High	**High**
Kim et al., 2011 [46]	Moderate	Moderate	Moderate	Moderate	Moderate	**Moderate**
Korkar et al., 2020 [47]	High	High	Moderate	Low	High	**High**
Vigren et al., 2020 [48]	Moderate	High	Moderate	Moderate	Moderate	**Moderate**
Minkin et al., 2021 [49]	Moderate	Moderate	Moderate	Moderate	Moderate	**Moderate**
Vigren et al., 2023 [50]	Moderate	Low	Moderate	Moderate	Moderate	**Moderate**
Uda et al., 2024 [51]	High	High	Moderate	Low	Moderate	**High**

Risk of bias was assessed using a modified Newcastle-Ottawa Scale [44] adapted for retrospective surgical case series. High = major methodological concerns, Moderate = some concerns but interpretable, Low = minimal concerns. Overall risk reflects the composite assessment across domains

## Discussion

### An overview

This systematic review of seven retrospective studies comprising 231 patients from six countries over three decades suggests that AC is feasible in carefully selected patients undergoing resection for drug-resistant non-tumor epilepsy involving or adjacent to eloquent cortex. The available evidence supports procedural feasibility and acceptable safety profiles in expert centers; however, it does not establish definitive efficacy or equivalence to surgery under general anesthesia.

Neurosurgical intervention can be an effective treatment for drug-resistant epilepsy [52, 53]; however, a major challenge is controlling seizures without causing significant neurological loss of function [54]. Patient selection is therefore central to awake epilepsy surgery. Accordingly, AC was primarily reserved for carefully selected patients able to cooperate with intraoperative mapping, whereas severe anxiety, cognitive impairment, severe dysphasia, morbid obesity, and major cardiopulmonary comorbidities were considered contraindications [55–61].

Successful epilepsy surgery requires accurate localization of both the epileptogenic zone and eloquent cortex. Although non-invasive and invasive investigations aid seizure localization, they cannot replace intraoperative functional mapping for identifying eloquent cortex and critical white matter pathways [47, 62–66].

Brain mapping remains fundamental during awake epilepsy surgery because functional reorganization and neuroplasticity are common, particularly in patients with early-onset epilepsy and developmental lesions, making anatomical landmarks less reliable predictors of functional organization [33, 48, 49, 67–69].

An important methodological consideration is the definition of non-tumor epilepsy. In clinical epilepsy surgery, developmental lesions such as focal cortical dysplasia and DNET differ substantially from diffuse gliomas because surgical decision-making is directed primarily toward seizure control rather than oncological management. Nevertheless, these lesions represent heterogeneous biological entities that may influence postoperative seizure outcomes. Because individual patient data were unavailable, pathology-specific subgroup analyses or exclusion of DNET patients were not feasible. Future multi-center studies should report seizure outcomes separately according to underlying pathology to facilitate more robust comparative analyses.

The pooled random-effects estimate demonstrated that 59.3% (95% CI 46.3–71.2%) of patients achieved Engel class I seizure freedom following awake craniotomy. This estimate should be interpreted cautiously because it represents pooled observational evidence derived from heterogeneous patient populations, operative strategies, and underlying pathologies. Importantly, this proportion does not represent treatment efficacy but rather a summary of reported outcomes across the available literature.

Published systematic reviews of conventional epilepsy surgery performed under general anesthesia for more homogeneous pathological entities, including mesial temporal lobe epilepsy and focal cortical dysplasia, have generally reported higher rates of postoperative seizure freedom, often ranging from approximately 60% to 80%, depending on pathology, duration of follow-up, and completeness of resection. However, these comparisons should be interpreted as descriptive rather than comparative. Patients undergoing awake craniotomy are typically selected because the epileptogenic zone is located within or adjacent to eloquent cortex, representing a surgically more challenging population with different operative priorities, particularly preservation of neurological function. Consequently, direct comparison of seizure outcomes between these cohorts is inappropriate and should not be interpreted as evidence of relative treatment effectiveness.

### Intraoperative seizures, stimulation parameters, and technical mapping considerations

Intraoperative seizures are a particularly relevant complication in awake epilepsy surgery because electrical stimulation is applied in or near epileptogenic cortex and may interrupt mapping, reduce patient cooperation, and alter the operative workflow. Across the included studies, reporting was inconsistent. Minkin et al. [49] reported intraoperative seizures in 4 of 14 AC patients (28.6%), Kim et al. [46] listed seizures among intraoperative complications without a fully extractable denominator, and several studies did not specify whether stimulation-related seizures occurred. Because preventive antiepileptic drug strategies, stimulation intensity, stimulation duration, afterdischarge monitoring, use of cold saline irrigation, and ECoG protocols were not consistently described, we could not validly compare seizure risk with awake tumor surgery or surgery under general anesthesia. Future studies should report stimulation parameters, afterdischarges, intraoperative seizure management, perioperative antiepileptic therapy, interruption of mapping, and whether seizures altered the planned extent of resection.

The birth of AC was associated with the resection of epileptogenic foci by Sir Victor Horsley [1]. Subsequently, AC was mainly associated with epilepsy neurosurgery in the early and mid-20th century [47, 70–73]. This was partially due to the availability of electrocorticography to identify epileptic foci as well as the effect of general anesthesia on electrocorticography in the early twentieth century [47]. During the past few decades, with the advancements in neuroanesthesia, neuroimaging techniques, presurgical investigations and neuronavigation, there has been a shift towards epilepsy surgery under general anesthesia relying more on preoperative planning, and AC has been spared for specific cases.

Although awake craniotomy is well established in glioma surgery, epilepsy presents distinct challenges because epileptogenic networks and functional reorganization may differ substantially from tumor-related anatomical distortion. These differences highlight the importance of individualized functional mapping during epilepsy surgery [42, 47, 63, 74–78].

Another major challenge in epilepsy surgery is the predictability of subcortical pathways in epilepsy neurosurgery. While subcortical pathways have been well-described in tumor neurosurgery [27, 79], the identification of these pathways in epilepsy is less understood [80, 81]. Therefore, further studies are encouraged to understand the development and localization of subcortical networks in epileptogenic malformations [48]. Therefore, preoperative planning can benefit from tractography to visualize important tracts, including the corticospinal tract, the optic radiation, the arcuate fasciculus and association pathways [48].

A long-held debate on the safety and efficacy of epilepsy surgery has focused on two common temporal lobe epilepsy methods: temporal lobe resection vs. amygdalahippocampectomy [82]. Preoperative memory lateralization assessment through Wada test or fMRI is common in identifying an appropriate surgical candidate for these procedures [83, 84]. The efficacy of intraoperative assessment of memory lateralization during AC using new technologies such as virtual reality needs to be explored [85–87].

In epilepsy, a resection that does not improve seizure control or enhance the quality of life is not motivated [48]. Therefore, postoperative rehabilitation is an important aspect of epilepsy surgery due to the risk of the “burden of normality” [88–92]. While resective surgery can achieve seizure control and preserve neuro-psychological functions, existing cognitive deficits, which were previously limited by epilepsy, can become more apparent once the patient faces normal challenges in life [48, 49, 88–91]. Although the efficacy of resection of epileptogenic regions with AC in controlling seizure is comparable to the ones done under GA [91–94], future large-scale controlled studies on patients with different seizure pathologies are required to compare the risk of postoperative neurological deficits in these two methods. Although the limited comparative studies reported similar postoperative seizure outcomes, these findings should be interpreted with considerable caution. Patients undergoing awake craniotomy frequently represent a highly selected subgroup with epileptogenic zones located within or adjacent to eloquent cortex, whereas patients undergoing surgery under general anesthesia often differ substantially with respect to lesion characteristics, surgical complexity, and operative objectives. Consequently, the available evidence is insufficient to determine whether awake craniotomy offers equivalent, superior, or inferior seizure outcomes compared with conventional surgery under general anesthesia. In addition, future studies can investigate the molecular mechanism of seizure by studying subcellular components and metabolic changes of the resected tissue [85].

Importantly, the existing comparative literature consists exclusively of retrospective observational studies without randomization or adjustment for important confounding variables. Selection bias is particularly relevant because awake craniotomy is generally reserved for patients in whom preservation of neurological function is a major surgical consideration [95]. Therefore, any apparent similarities or differences in seizure outcomes between awake craniotomy and general anesthesia may reflect differences in patient selection rather than the anesthetic or surgical approach itself.

### Limitations

Our systematic review has several limitations. All included studies were retrospective, and most were small single-center series, resulting in moderate to high risks of selection, reporting, and confounding bias. Although the review was not prospectively registered, the eligibility criteria, search strategy, study selection, data extraction, and quality assessment were predefined and conducted in accordance with PRISMA recommendations. The included cohorts were clinically heterogeneous with respect to patient characteristics, pathology, lesion location, mapping strategies, anesthetic protocols, and follow-up duration. Pediatric and adult outcomes could not be analyzed separately because mixed-age studies did not report age-stratified data. Although a single-arm random-effects analysis of Engel class I seizure freedom was performed, differences in study design and outcome reporting preclude conclusions regarding efficacy or equivalence with surgery under general anesthesia. In addition, inconsistent reporting of intraoperative seizures, stimulation parameters, perioperative antiepileptic therapy, quality of life, and psychosocial outcomes limited quantitative synthesis. Finally, inclusion of heterogeneous developmental epileptogenic lesions without patient-level data precluded pathology-specific analyses, and restriction to English-language publications may have introduced publication bias. Despite these limitations, this review provides a focused synthesis of the available evidence and highlights priorities for future research.

## Conclusion

Based on the currently available retrospective evidence, AC with intraoperative functional mapping appears feasible for selected patients with drug-resistant non-tumor epilepsy involving or adjacent to eloquent cortex, particularly when functional preservation is critical. Reported seizure outcomes and neurological morbidity are encouraging, but the evidence is heterogeneous and insufficient to establish definitive efficacy, superiority, or equivalence to surgery under general anesthesia. Current evidence does not permit conclusions regarding the comparative effectiveness of awake craniotomy versus surgery under general anesthesia. Clinicians considering AC for epilepsy should engage in multidisciplinary discussion, ensure adequate patient selection, and counsel patients regarding the limited comparative evidence. Future prospective multicenter studies should use standardized reporting of patient selection, stimulation parameters, perioperative antiepileptic strategies, intraoperative seizures, mapping reliability, seizure outcomes, neurological deficits, quality of life, and psychosocial adaptation.

## Supplementary Information

Supplementary Table 1: An overview of search terms used for each database.

## Data Availability

The datasets used and/or analysed during the current study are available from the corresponding author on reasonable request.

## Abbreviations

AC: Awake craniotomy
AED: Antiepileptic drug
CI: Confidence interval
CPS: Complex partial seizure(s)
DNET: Dysembryoplastic neuroepithelial tumor
ECoG: Electrocorticography
EEG: Electroencephalography
Engel: Engel seizure outcome classification
FAS: Focal aware seizure
FBTCS: Focal to bilateral tonic–clonic seizure
FCD: Focal cortical dysplasia
FDG: PET–Fluorodeoxyglucose positron emission tomography
FIAS: Focal impaired awareness seizure
fMRI: Functional magnetic resonance imaging
GA: General anesthesia
I²: Higgins’ inconsistency statistic (heterogeneity statistic)
MEG: Magnetoencephalography
MRI: Magnetic resonance imaging
NA: Not applicable
NOS: Newcastle–Ottawa Scale
NS: Not specified
PET: Positron emission tomography
PRISMA: Preferred Reporting Items for Systematic Reviews and Meta–Analyses
SD: Standard deviation
SPECT: Single–photon emission computed tomography
UK: United Kingdom
USA: United States of America
