# Supplementary material for: Awake craniotomy for drug-resistant non-oncological epilepsy: a systematic review of feasibility, reported safety, and seizure outcomes
Source: BMC Surg. 2026 Aug 10;26:751. doi: 10.1186/s12893-026-04115-4 (PMC13737535; doi:10.1186/s12893-026-04115-4)
Supplement: Supplementary file 1 [file 12893_2026_4115_MOESM1_ESM.docx]

**Supplementary Table 1.** An Overview of Search Terms Used for Each Database

| **Database** | **Search Results** | **Search Terms** |
| --- | --- | --- |
| **PubMed** | 4736 | (("awake"[All Fields] OR "awakeness"[All Fields] OR "awakes"[All Fields] OR "awaking"[All Fields]) AND ("craniotomy"[MeSH Terms] OR "craniotomy"[All Fields] OR "craniotomies"[All Fields])) OR (("awake"[All Fields] OR "awakeness"[All Fields] OR "awakes"[All Fields] OR "awaking"[All Fields]) AND ("brain"[MeSH Terms] OR "brain"[All Fields] OR "brains"[All Fields] OR "brain s"[All Fields]) AND ("surgery"[MeSH Subheading] OR "surgery"[All Fields] OR "surgical procedures, operative"[MeSH Terms] OR ("surgical"[All Fields] AND "procedures"[All Fields] AND "operative"[All Fields]) OR "operative surgical procedures"[All Fields] OR "general surgery"[MeSH Terms] OR ("general"[All Fields] AND "surgery"[All Fields]) OR "general surgery"[All Fields] OR "surgery s"[All Fields] OR "surgerys"[All Fields] OR "surgeries"[All Fields])) OR (("awake"[All Fields] OR "awakeness"[All Fields] OR "awakes"[All Fields] OR "awaking"[All Fields]) AND ("neurosurgery"[MeSH Terms] OR "neurosurgery"[All Fields] OR "neurosurgeries"[All Fields] OR "neurosurgery s"[All Fields] OR "neurosurgical procedures"[MeSH Terms] OR ("neurosurgical"[All Fields] AND "procedures"[All Fields]) OR "neurosurgical procedures"[All Fields])) OR (("awake"[All Fields] OR "awakeness"[All Fields] OR "awakes"[All Fields] OR "awaking"[All Fields]) AND ("brain mapping"[MeSH Terms] OR ("brain"[All Fields] AND "mapping"[All Fields]) OR "brain mapping"[All Fields]) AND ("awake"[All Fields] OR "awakeness"[All Fields] OR "awakes"[All Fields] OR "awaking"[All Fields]) AND ("cysts"[MeSH Terms] OR "cysts"[All Fields] OR "cyst"[All Fields] OR "neurofibroma"[MeSH Terms] OR "neurofibroma"[All Fields] OR "neurofibromas"[All Fields] OR "tumor s"[All Fields] OR "tumoral"[All Fields] OR "tumorous"[All Fields] OR "tumor"[All Fields] OR "neoplasms"[MeSH Terms] OR "neoplasms"[All Fields] OR "tumor"[All Fields] OR "tumors"[All Fields] OR "tumoral"[All Fields] OR "tumors"[All Fields] OR "tumors"[All Fields] OR "tumors"[All Fields]) AND ("resect"[All Fields] OR "resectability"[All Fields] OR "resectable"[All Fields] OR "resectates"[All Fields] OR "resected"[All Fields] OR "resecting"[All Fields] OR "resection"[All Fields] OR "resectional"[All Fields] OR "resectioned"[All Fields] OR "resectioning"[All Fields] OR "resections"[All Fields] OR "resective"[All Fields] OR "resects"[All Fields])) OR (("craniotomy"[MeSH Terms] OR "craniotomy"[All Fields] OR "craniotomies"[All Fields]) AND ("awake"[All Fields] OR "awakeness"[All Fields] OR "awakes"[All Fields] OR "awaking"[All Fields])) |
| **Scopus** | 5840 | ALL ( awake AND craniotomy OR awake AND brain AND surgery OR awake AND neurosurgery OR awake AND brain AND mapping AND awake AND tumor AND resection OR craniotomy AND while AND awake) |
| **Web of Science** | 4377 | ALL=(awake craniotomy OR awake brain surgery OR awake neurosurgery OR awake brain mapping OR awake tumor resection OR craniotomy while awake) |
